# A longer time to relapse is associated with a larger increase in differences between paired primary and recurrent IDH wild-type glioblastomas at both the transcriptomic and genomic levels

**DOI:** 10.1186/s40478-024-01790-3

**Published:** 2024-05-18

**Authors:** Wei-Min Ho, Chia-Ying Chen, Tai-Wei Chiang, Trees-Juen Chuang

**Affiliations:** 1 Genomics Research Center, Academia Sinica, Taipei, Taiwan; 2 Ph.D. Program in Translational Medicine, National Taiwan University and Academia Sinica, Taipei, Taiwan; 3 Department of Neurology, Chang Gung Memorial Hospital, Linkou Medical Center, Taoyuan, Taiwan; 4 College of Medicine, Chang Gung University, Taoyuan, Taiwan; 5 School of Medicine, National Tsing Hua University, Hsinchu, Taiwan

**Keywords:** Glioblastomas, Patient-matched longitudinal analysis, Time to relapse, Prognostic model, Progression-free survival

## Abstract

Glioblastoma (GBM) is the most common malignant brain tumor in adults, which remains incurable and often recurs rapidly after initial therapy. While large efforts have been dedicated to uncover genomic/transcriptomic alternations associated with the recurrence of GBMs, the evolutionary trajectories of matched pairs of primary and recurrent (P-R) GBMs remain largely elusive. It remains challenging to identify genes associated with time to relapse (TTR) and construct a stable and effective prognostic model for predicting TTR of primary GBM patients. By integrating RNA-sequencing and genomic data from multiple datasets of patient-matched longitudinal GBMs of isocitrate dehydrogenase wild-type (IDH-wt), here we examined the associations of TTR with heterogeneities between paired P-R GBMs in gene expression profiles, tumor mutation burden (TMB), and microenvironment. Our results revealed a positive correlation between TTR and transcriptomic/genomic differences between paired P-R GBMs, higher percentages of non-mesenchymal-to-mesenchymal transition and mesenchymal subtype for patients with a short TTR than for those with a long TTR, a high correlation between paired P-R GBMs in gene expression profiles and TMB, and a negative correlation between the fitting level of such a paired P-R GBM correlation and TTR. According to these observations, we identified 55 TTR-associated genes and thereby constructed a seven-gene (*ZSCAN10*, *SIGLEC14*, *GHRHR*, *TBX15*, *TAS2R1*, *CDKL1*, and *CD101*) prognostic model for predicting TTR of primary IDH-wt GBM patients using univariate/multivariate Cox regression analyses. The risk scores estimated by the model were significantly negatively correlated with TTR in the training set and two independent testing sets. The model also segregated IDH-wt GBM patients into two groups with significantly divergent progression-free survival outcomes and showed promising performance for predicting 1-, 2-, and 3-year progression-free survival rates in all training and testing sets. Our findings provide new insights into the molecular understanding of GBM progression at recurrence and potential targets for therapeutic treatments.

**Supplementary Information:**

The online version contains supplementary material available at 10.1186/s40478-024-01790-3.

## Introduction

Glioblastoma (GBM), which was classified as grade 4 glioma by the World Health Organization (WHO), is the most common and aggressive malignant brain tumor in adults [1, 2]. The registry data from 2000 to 2014 in the US revealed that the overall 5-year survival rate of GBMs is only 5.4% [3]. It was observed that the vast majority (90%) of GBMs were primary arising *de novo* (or newly diagnosed GBMs) [4]; hence, most GBM preclinical studies are based on tissues from primary GBMs. The standard treatment of primary GBMs consists of surgical tumor resection followed by radiotherapy plus continuous daily temozolomide (TMZ) [5, 6]. However, the overall effectiveness of such a treatment is limited because of the resistance to TMZ or radiotherapy [7] and the postoperative GBM recurrence [8]. GBMs often recur rapidly after initial therapy; approximately 90% of patients were affected by GBM recurrence [9]. Unfortunately, no effective therapeutic protocol for the tumor recurrence is available currently, leading to an extremely poor prognosis [5, 10]. Numerous studies have attributed the treatment failure of relapsed GBMs to the high degree of intratumoral spatial heterogeneity among GBM patients [11–15]. The heterogeneity of treatment outcomes and interactions between various clinical factors complicate the accurate and reliable predictions of the time to relapse (TTR) for patients with primary GBM, hampering timely diagnosis and treatment for the patients. Numerous studies have investigated genetic and transcriptomic alternations associated with the recurrence of GBMs [16–23], greatly increasing our understanding of the evolutionary trajectories of paired primary-recurrent (P-R) GBMs. However, the relationships of TTR to the differences between paired P-R GBMs at both the transcriptomic and genomic levels remain largely elusive. Analyses of such relationships may help identifying genes associated with long-term progression-free survival (PFS), an important response measure for primary GBM patients, and then evaluate the effects of present treatments/therapies on disease burden and health-related quality of life [24, 25]. Meanwhile, investigation of longitudinal changes in gene expression may provide clues for the intratumoral spatial heterogeneity and identification of new therapeutic and predictive biomarkers of malignant progression, shedding light on GBM etiology and recurrence.

A major challenge to achieve the abovementioned goal is the limited sample size for transcriptomic and genomic data from paired P-R GBM specimens. Recurrent GBMs are not always biopsied, not to mention the difficulty in acquiring omics data from both paired P-R GBM samples. For example, regarding the RNA-seq data of GBM patients in the Cancer Genome Atlas (TCGA; http://cancergenome.nih.gov/), less than 8% of samples are derived from recurrent tumors and the primary and recurrent samples are mostly not biopsied from the same patients. Covariate adjustment has been another challenge for analyses of differences between conditions. Covariates encompass a wide range of technical and biological factors, such as data source, library preparation batch, age, gender, WHO grade, and isocitrate dehydrogenase (IDH) status, which often exert varying degrees of influence on gene expression and confound the interpretation of the observed differences between conditions [26]. Some covariates such as IDH status, age, and gender were demonstrated to considerably impact the GBM patient outcome in prognostic models for survival prediction [27]. Adjusting for survival-related covariates can improve the statistical power and minimize potential false positives arising from biological bias or technical artifacts [28]. However, missing values ​​often occur in the available clinical information, catching in a dilemma of whether to sacrifice sample size in order to account for some covariates.

The newly released dataset generated by the Glioma Longitudinal Analysis Consortium (GLASS) [29], which comprises a large sample size of genomic data, RNA-seq data, and the corresponding clinical information from patient-matched longitudinal glioma samples, offers an unprecedented opportunity for investigating the evolutionary trajectories of paired P-R GBMs in heterogeneities at both the transcriptomic and genomic levels. We thus examined the following issues: (i) the effects of various covariates on gene expression; (ii) the association of TTR with transcriptomic/genomic differences between paired P-R GBMs; (iii) the association of TTR with cellular heterogeneity between paired P-R GBMs; (iv) the correlation between paired P-R GBMs in gene expression profiles and tumor mutation burden (TMB); and (v) the relationship of TTR to the P-R correlation observed in (iv). On the basis of the results of the above analyses, we identified 55 TTR-associated genes and utilized the 55 genes to subdivide the primary GBM patients into two molecular subtypes. Our results revealed that these two subtypes represented significant differences in TTR and outcomes of PFS. We further constructed a seven-gene prognostic classifier for predicting TTR of primary GBM patients. We demonstrated that the constructed model had promising accuracy and sensitivity in all the training set (the GLASS cohort) and two independent testing sets. Overall, this study unveiled the association of TTR with differences between paired P-R GBMs at both the genomic and transcriptomic levels and constructed a stable prognostic model for PFS prediction, contributing to our understanding of the GBM development and progression at recurrence and the potential for therapeutic treatments.

## Methods

### Data collection and preprocessing

**Fig. 1:**
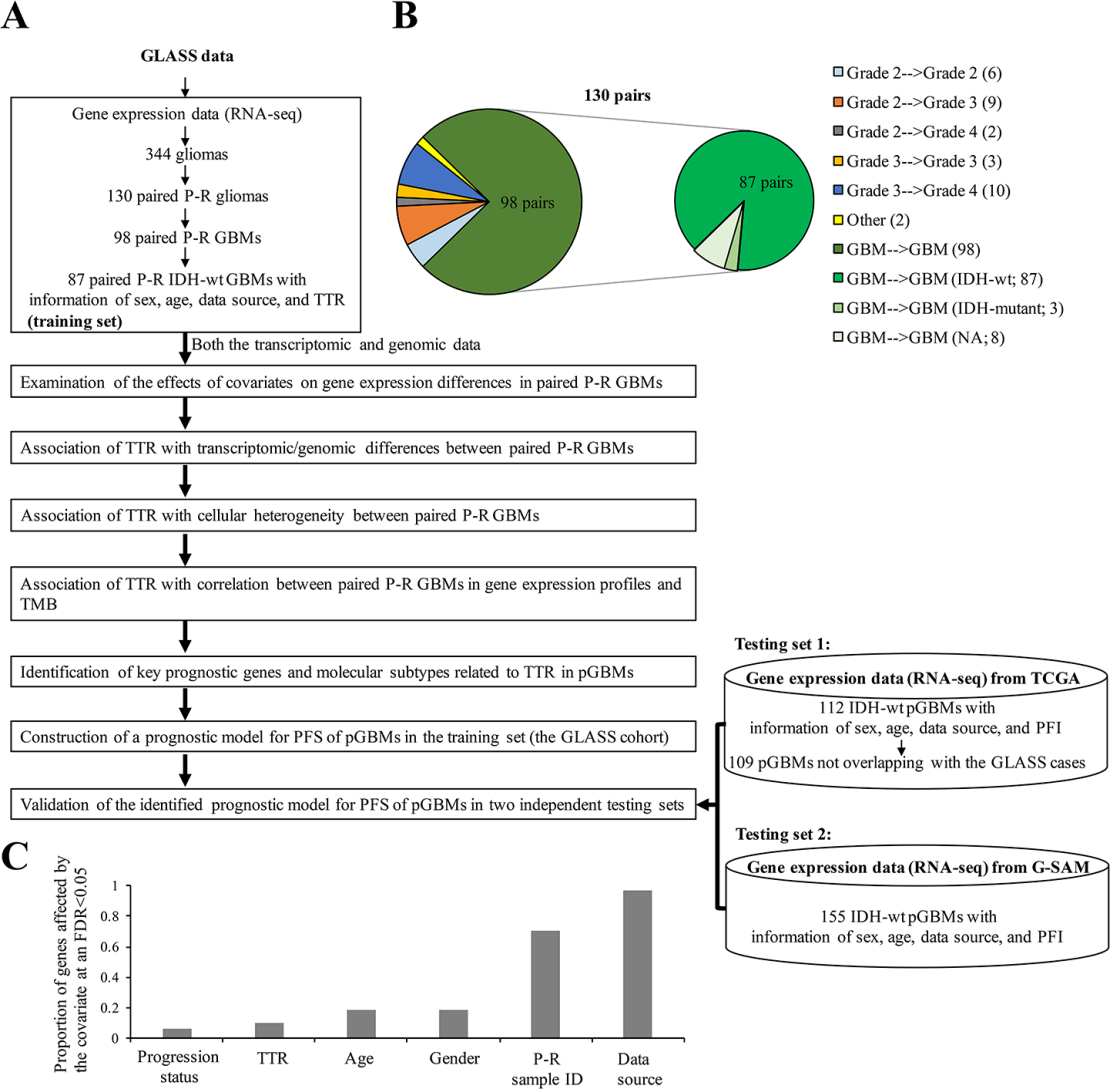
Investigation of the differences between paired P-R GBMs at both the transcriptomic and genomic levels. **A** Summary of the workflow for data extractions, analyses, and prognostic model construction in this study. pGBM, primary GBM. **B** Distribution of various types of paired P-R gliomas. Numbers in parentheses indicated the number of glioma pairs. NA, IDH status unavailable. **C** Distribution of the proportions of genes affected by various covariates at an FDR < 0.05

Gene expression profile (transcripts per kilobase million or TPM), mutation calls, and the corresponding clinical information of the GLASS glioma samples [29] were downloaded from Synapse (https://www.synapse.org/#!Synapse:syn26465623), respectively. Genome assembly GRCh38 and Ensembl gene annotation (version 100) were employed for the analyses. As shown in Fig. 1A and B and 87 patient-matched longitudinal samples with both primary and recurrent (surgery 1 and surgery 2) IDH wild-type (IDH-wt) GBMs were considered in this study (Additional file 1: Table S1). To minimize potential spurious events, only the genes expressed (i.e., TPM > 0) in > 50% of the examined GBM samples (174 primary or recurrent GBM samples) were retained, leaving 21,475 gene expression features (Additional file 2: Table S2). The expression levels of genes were measured by the log_2_ (TPM + 1) values with regressing out the effects of the four covariates (i.e., age, gender, data source, and paired P-R sample IDs) using a linear model (the lm() function in the R package). The various types of gene mutation features were downloaded from the GLASS data table at https://www.synapse.org/#!Synapse:syn17038081/tables/. Only the variant event whose read count of the alternative allele was greater than two was considered. TMB (mutations/Mb) values were downloaded from the GLASS data table at https://www.synapse.org/#!Synapse:syn32908224/tables/ (see also Additional file 3: Table S3). As for the TCGA dataset, TPM and the corresponding clinical information of GBM samples were downloaded from GDC (https://portal.gdc.cancer.gov/). A total of 109 primary IDH-wt GBMs that did not overlap with the GLASS cases and had all the corresponding clinical information of age, gender, data source, and progression-free interval (PFI) (see Fig. 1A and Additional file 4: Table S4) were considered as a testing set for assessing the prediction power of the constructed seven-gene prognostic model. The expression levels of genes were measured by the log_2_ (TPM + 1) values with regressing out the effects of age, gender, and data source using a linear model. An additional testing set, The Glioblastoma, Stability of Actionable Mutations (G-SAM) dataset [30], was downloaded from The European Genome-phenome Archive (https://ega-archive.org/) with permission under the accession number of EGAD00001007860. A total of 155 primary IDH-wt GBMs with RNA-seq data and the corresponding clinical information of age, gender, data source, and PFI were downloaded for validation (Fig. 1A and Additional file 4: Table S4). The clinical information was obtained from the authors of the G-SAM study [30]. RNA-seq data from more than one sequencing run of the same sample was merged. The TPM values were calculated using the RSEM tool [31] based on the STAR [32] alignment results. Similarly, the expression levels of genes were measured by the log_2_ (TPM + 1) values with regressing out the effects of age, gender, and data source.

### Covariate assessment and differential expression analysis

We employed a linear regression model (i.e., the lm() function in the R package) to assess the effect of each of the six covariates including progression status (primary or recurrence), TTR, age, gender, data source, and paired P-R sample ID on gene expression. The effect of a covariate was measured by the proportion of genes affected by this covariate at a false discovery rate (FDR) < 0.05 when adjusting for the other five covariates (Fig. 1C and Additional file 1: Table S1). The *P* values were FDR-adjusted using Benjamini-Hochberg correction. The lm() function was utilized to identify differentially expressed genes (DEGs) between paired P-R GBM samples for all patients and patients in each of the three TTR groups (i.e., TTR ≤ 6 months, TTR of 7–12 months, and TTR < 12 months), respectively (Additional file 2: Table S2).

### Bulk transcriptional subtype classification and deconvolution analysis

On the basis of the adjusted gene expression levels stated above, we evaluated the representation of the classical, proneural, and mesenchymal bulk transcriptional subtypes across the GLASS dataset using the “ssgsea.GBM.classification” R package [33]. For each GBM sample, the subtype with the lowest *P* value was assigned to this sample. If there was more than one subtype with the lowest *P* value, the subtype with the highest enrichment score was assigned to this sample. The CIBERSORTx web tool (https://cibersortx.stanford.edu/) [34] in conjunction with the covariate-adjusted gene expression levels was utilized to estimate the cellular proportions of the 12 types of cell states for each GLASS sample with batch correction of bulk model, disabling quantile normalization, absolute model, and permutations = 1000. The signature matrix file, which was generated by two single-cell RNA-seq datasets [35, 36], was downloaded from the GLASS study [29].

### Assessment of correlation between paired P-R GBMs in gene expression profiles and TMB

The correlations between paired P-R GBMs in gene expression profiles or TMB (mutations/Mb) were assessed using the Pearson’s correlation test. For the CLASS cohort [29] (87 paired P-R IDH-wt GBMs), the expression levels of genes were measured by the log_2_ (TPM + 1) values with regressing out the effects of age, gender, and data source using a linear model (the lm() function in the R package). For another dataset with 16 paired P-R IDH-wt GBMs (i.e., Korber et al.’s dataset [19]), the RNA-seq data were downloaded from the European Genome-phenome Archive with permission under the accession number of EGAD00001004564. The TPM values were calculated using the same procedures stated above. The expression levels of genes were measured by the log_2_ (TPM + 1) values with regressing out the effect of gender (the information of age was not publicly accessible). The transcriptomic similarity between GBM samples was measured by the transcriptome overlap measure (TROM) scores using the TROM method in the R package on CRAN (https://github.com/Vivianstats/TROM) [37]. A higher TROM score indicates a higher level of transcriptomic similarity between samples. The heatmap was created using the heatmap() function in the R package. The gene expression profile for each GBM sample was measured by the PC1 (principal component 1) value of all genes in the examined sample through the principal component analysis (PCA) (Additional file 3: Table S3). The single-cell RNA-seq data from 30 paired P-R GBM samples were publicly downloadable from the NCBI Gene Expression Omnibus repository (https://www.ncbi.nim.nih.gov/geo/) with the accession number of GSE174554 [38] and processed by the Seurat package (version 5.0.1) [39]. The ReadMtx function was utilized to read in the expression matrix of each sample. Each Seurat object was created by CreateSeurateObject function. Here we considered the paired P-R GBMs if the samples were IDH-wt tumors and simultaneously had TTR information and the CreateSeurateObject parameters with min.cells > 150 and min.features > 200. After that, 13 paired P-R GBMs were retained in our analyses. Each sample object was normalized by the NormalizedData function. Variable genes were identified by the FindVariableFeatures function with default parameters. The ScaleData function was employed to scale the integrated object and then determined 1,710 variable features. The top 10 PCs were determined by the RunPCA function and calculated by the ProjecteDim function. For each sample, we extracted the PC values using the SplitObject function and retained the PC1 value for each variable feature (gene). For each gene, the PC1 value, which represented the expression level of this gene, was adjusted with regressing out the effects of age and gender using the linear model abovementioned. Like the process stated above, the gene expression profile for each GBM sample was measured by the PC1 value of all the 1,710 genes in this sample through the PCA analysis. The linear regression equation and the fitting line were conducted using the lm() function. The fitting levels of the observed data in an examined TTR group were measured by the root mean square error (RMSE) values. Using Fig. 4B as an example, the RMSE value for the cases with TTR ≤ 6 months (*n* = 23) was calculated by

$$\text{R}\text{M}\text{S}\text{E}=\sqrt{\frac{1}{n}\sum _{i=1}^{n}({{y}_{i}-\widehat{{y}_{i}})}^{2}}$$

where *y*_*i*_ and $\widehat{{y}_{i}}$ were PC1 of the recurrent GBM case *i* and *β*_*0*_ + *β*_*1*_×PC1 of the primary GBM case *i*, respectively. *β*_*0*_ and *β*_*1*_ represented the intercept and slope of the linear regression equation (Y=-2.546 × 10^− 16^+0.448X; see Fig. 4A), respectively. The tumor hypermutation status of the G-SAM dataset was obtained from the authors of the G-SAM study [30] (see also Additional file 4: Table S4).

### Identification of TTR-associated genes

We identified TTR-associated genes if the genes simultaneously satisfied the following criteria. First, the genes were not expressed significantly differentially (*P* > 0.05 by the differential expression analysis stated above) between paired P-R GBMs in the patients with TTR ≤ 6 months. Second, the gene expression levels of the paired P-R GBMs were highly correlated with each other (*P* < 0.05 by Pearson’s correlation test). Third, the genes should pass both the Kaplan-Meier analysis and the univariate Cox proportional hazards regression analysis (both *P* < 0.05). Fourth, only the protein-coding genes expressed in both the training (GLASS) and testing (TCGA) sets were considered. After that, 55 TTR-associated protein-coding genes were identified (see Additional file 2: Table S2). The signaling pathway analyses (KEGG- and Hallmark-based pathways) and the Gene Ontology (GO) analysis were performed by modEnrichr (https://maayanlab.cloud/modEnrichr/) [40]. The ConsensusClusterPlus analysis [41] was performed to identify different molecular groups in the primary GBM patients (87 cases; the GLASS dataset).

### Construction of prognostic models for PFS

The multivariate stepwise regression analysis was employed to identify the robust independent gene signature for PFS prediction in primary IDH-wt GBM patients. On the basis of the 55 TTR-associated protein-coding genes mentioned in the previous section, the prognostic model (Model-ours) with seven key genes (*SIGLEC14*, *GHRHR*, *TAS2R1*, *CDKL1*, *ZSCAN10*, *TBX15*, and *CD101*; Additional file 4: Table S4) was thereby constructed using the coxph function. A patient’s risk score was evaluated by the predictive function of the survival R package. The mathematical formula was:

$$Risk score = \text{exp}\left(\sum _{i=1}^{7}{coefficient}_{i}\times {expression}_{i}\right)$$

where *coefficient*_*i*_ and *expression*_*i*_ represented the regression coefficient and the covariate-adjusted expression level of the *i*th gene, respectively. The coefficients of *SIGLEC14*, *GHRHR*, *TAS2R1*, *CDKL1*, *ZSCAN10*, *TBX15*, and *CD101* were 0.3296, 0.2804, 2.1475, -0.7833, 0.8261, 0.5557, and − 0.4108, respectively. The patients were divided into high- and low-score groups according to the median risk score (1.04) of the 87 primary IDH-wt GBM cases in the GLASS cohort.

For Model-new1 construction, TTR-associated genes were identified if the genes simultaneously satisfied: (1) the genes were DEGs between paired P-R GBMs in the GLASS cohort (FDR < 0.05; Additional file 2: Table S2); and (2) the third and fourth rules stated in the previous section. Model-new1 with four prognostic markers was then constructed through a multivariate stepwise regression analysis (Additional file 4: Table S4). For Model-new2 construction, TTR-associated genes were identified if the genes simultaneously satisfied: (1) the gene expression levels of the primary GBMs were highly correlated with the PFI values (absolute value of Pearson’s correlation coefficient (*r*) ≥ 0.2 with *P* < 0.01 according to a previous study [42]); and (2) the third and fourth rules stated in the previous section. Model-new2 with five prognostic markers was constructed through a multivariate stepwise regression analysis (Additional file 4: Table S4).

### Statistical analysis

**Fig. 2:**
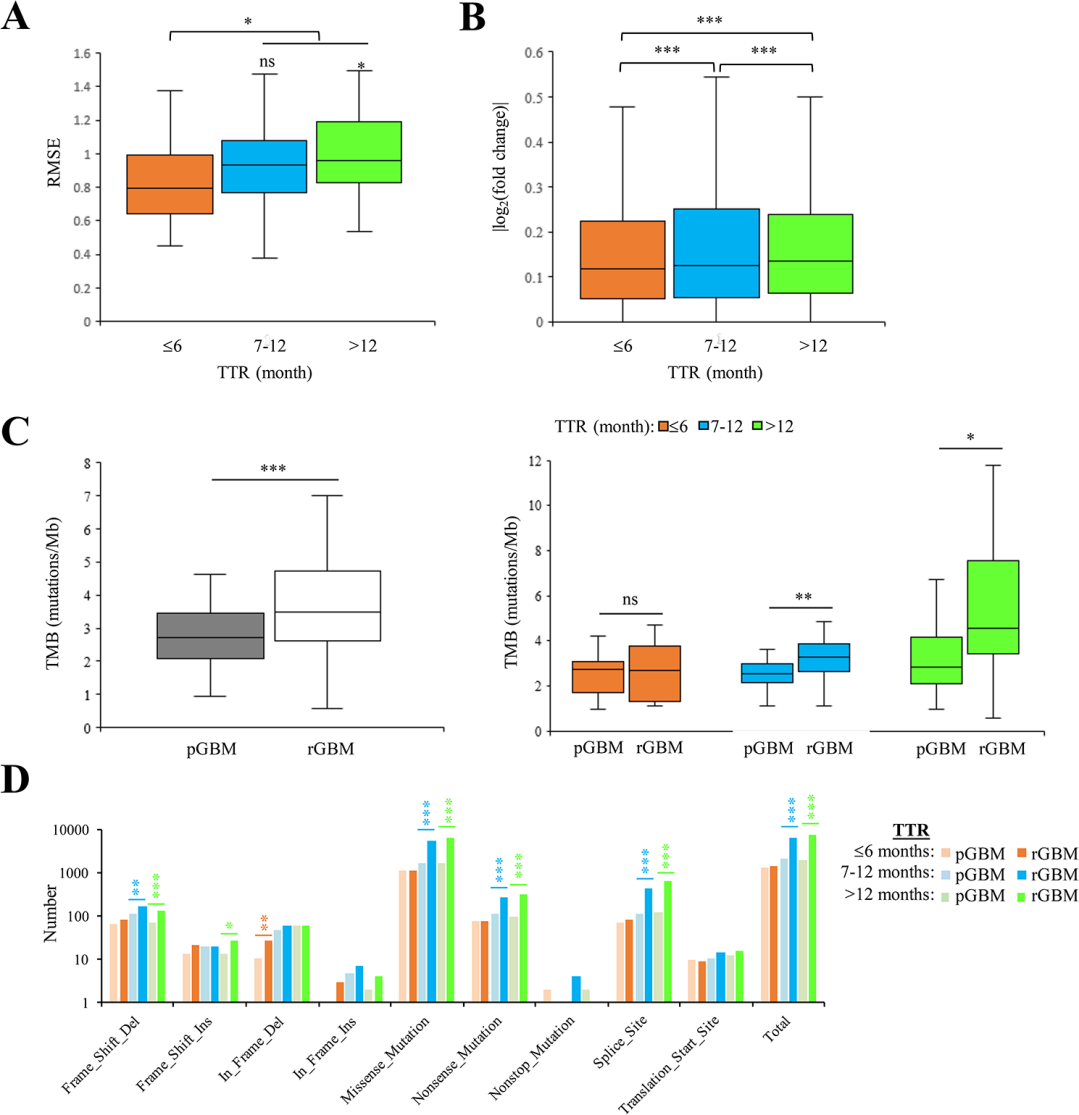
Associations of TTR with the differences between paired P-R GBMs at the transcriptomic and genomic levels. **A-B** Comparisons of the gene expression differences (measured by the RMSE) (**A**) and the effects of differential expression (measured by the absolute value of log2 transformed fold change) (**B**) between paired P-R GBMs for patients in the three TTR groups: patients with TTR ≤ 6 months, TTR of 7–12 months, and TTR < 12 months. **C** Comparisons of the TMB values (mutations/Mb) between paired P-R GBMs for all patients (left) and patients in the three TTR groups (right). **D** Comparisons of various types of gene mutation features for primary and recurrent GBMs in the three TTR groups. For (**A**)-(**C**), *P* values were evaluated using Wilcoxon signed rank test. For (**D**), *P* values were evaluated using one proportion Z-Test. pGBM, primary GBM. rGBM, recurrent GBM

All box and whisker plots represented median, quartiles (box), and range (whiskers). Correlation coefficients and *P* values were evaluated using the Pearson’s correlation analysis. For Fig. 2D, for each type of gene mutation features, we calculated the ratio of the mutation count in pGBMs to those in rGBMs and assessed whether this ratio was significantly different from 1 using one proportion Z-Test. To test whether the expression profiles of primary samples and their matched recurrent ones were highly similar, we conducted a permutation test to estimate *P* value for the paired P-R GBMs (e.g., 87 paired P-R GBMs in the GLASS dataset examined). In Fig. 4A, we randomly selected the equal number (i.e., 87) of paired GBM samples from all the 174 GBM samples (87 × 2) in the GLASS dataset (Additional file 3: Table S3). For each pair, the two selected samples were not the same. We calculated the TROM score for each pair and then the median value of TROM scores for the selected 87 paired samples. The process was performed 10,000 times and the *P* value was assessed. In Fig. 4B and C, we conducted a permutation test to estimate *P* value for each of the three TTR groups (i.e., TTR ≤ 6 months, TTR of 7–12 months, and TTR < 12 months). Taking the group of TTR ≤ 6 months (*n* = 23) as an example, we randomly selected the equal number of samples (i.e., *n* = 23) from the GLASS dataset and then calculated the RMSE value. The process was performed 10,000 times and the *P* value was assessed. The Kaplan-Meier analysis with the log-rank test was applied to assessing the statistical significance of PFS curves. A Cox proportional hazards model was employed for univariate and multivariate analyses to assess the gene expression on PFS outcomes. The multivariate model was constructed using the bidirection stepwise selection with both parameters of “sle” = 0.05 and “sls” = 0.05 (the stepwiseCox function in StepReg R package). The receiver operating characteristic curves (ROC) was performed using the roc.curve() function in the PRROC R package. The nomogram model and calibration plot were conducted using the rms R package. The *P* values of multiple comparisons were adjusted by the FDR with Benjamini-Hochberg correction. The statistical analyses used in the figures were also indicated in the corresponding figure legends. The one proportion Z-Test [43] was performed at https://www.medcalc.org/calc/test_one_proportion.php. The other statistical analyses were conducted using the R package of version 4.2.3.

## Results

### Normalization of gene expression and assessment of covariate effects

We extracted the gene expression data of patient-matched longitudinal samples and the corresponding clinical information from the GLASS cohort [29]. To investigate the effect of TTR on transcriptomic/genomic differences between paired P-R gliomas, we focused on the paired P-R gliomas in the GLASS dataset (Fig. 1A). The publicly downloadable GLASS dataset comprised 130 glioma patients with RNA-seq data for two time points (i.e., primary and first recurrence), of which more than 75% of them were GBM cases (i.e., progression from primary GBM to relapsed GBM; 98 cases) (Fig. 1B). Of the 98 patients, 87 were IDH-wt GBM cases. Since the sample size of the paired P-R GBMs with IDH-mutant was extremely limited, the 87 pairs of IDH-wt GBMs with complete information of sex, age, data source, and TTR were considered in this study (Fig. 1B and Additional file 1: Table S1). We asked whether some technical and biological confounding factors could considerably affect the analysis of DEGs between primary and recurrent GBM samples. We then employed a linear regression model to evaluate the effects of the covariates, including longitudinal progression status (primary or recurrent GBMs), age, gender, data source, TTR, and paired P-R sample IDs, on each gene (see Methods). The effect of a covariate on gene expression was evaluated by measuring the proportion of genes affected by this factor at an FDR < 0.05 when adjusting for the other covariates examined (Additional file 1: Table S1). Our results revealed that all the examined covariates can considerably impact gene expression (Fig. 1C). Importantly, we found that the effects of age, gender, paired P-R sample IDs, and data source (paired P-R sample IDs and data source particularly) were much higher than the effects of longitudinal progression status and TTR on gene expression (Fig. 1C). Thus, to examine the relationship between TTR and gene expression differences in paired P-R GBMs, we calculated the expression levels of genes with adjusting for the effects of age, gender, data source, and paired P-R sample IDs.

### Association of TTR with transcriptomic/genomic differences between paired primary-recurrent GBMs

We first calculated the RMSE values to evaluate the gene expression difference between each P-R GBM pair (Additional file 1: Table S1). We divided the 87 paired P-R GBMs into three groups according to TTR: 23 patients with TTR ≤ 6 months; 33 patients with TTR of 7–12 months; and 31 patients with TTR > 12 months. Our result showed that the RMSE values were generally lower in patients with TTR ≤ 6 months than in those with TTR > 6 months, suggesting a positive correlation between the gene expression differences and TTR (Fig. 2A). Next, we performed differential expression analyses between paired P-R GBMs for each of the three TTR groups mentioned above (Methods; Additional file 2: Table S2). Similarly, the effects of the transcriptional differences (i.e., the absolute value of log2 transformed fold change) significantly increased with increasing TTR values (Fig. 2B). Regarding TMB (e.g., mutations/Mb) of the paired P-R GBM patients, we observed that the TMB was significantly higher in recurrent GBM samples than in primary GBM samples (*P* = 0.00005 by Wilcoxon signed rank test; Fig. 2C, left). For the recurrent GBM samples, we also observed a positive correlation between the TTR and the TMB (Fig. 2C, right), consistent with the previous study [44]. Intriguingly, the significant differences in the TMB between the paired P-R GBM samples were observed for patients with a long TTR (e.g., TTR > 6 months), not for those with a short TTR (TTR ≤ 6 months) (Fig. 2C, right). Furthermore, we examined the counts of various types of gene mutation features in primary and recurrent GBMs among the three TTR groups (Additional file 1: Table S1). Consistently, the total mutation count was significantly larger in recurrent GBMs than in primary GBMs for patients with TTR > 6 months, not for those with TTR ≤ 6 months (Fig. 2D). Such significant differences were particularly found in the mutation features that could cause nonsynonymous changes or gain/loss of protein-coding nucleotides (e.g., deletion frameshift mutations, missense mutations, nonsense mutations, and mutations at splice sites; Fig. 2D). Taken together, these results revealed that both transcriptomic and genomic differences in paired P-R GBMs were remarkably lower in patients with a short recurrence interval as compared with those with a long recurrence interval.

### Association of TTR with cellular heterogeneity between primary and recurrent GBMs

**Fig. 3:**
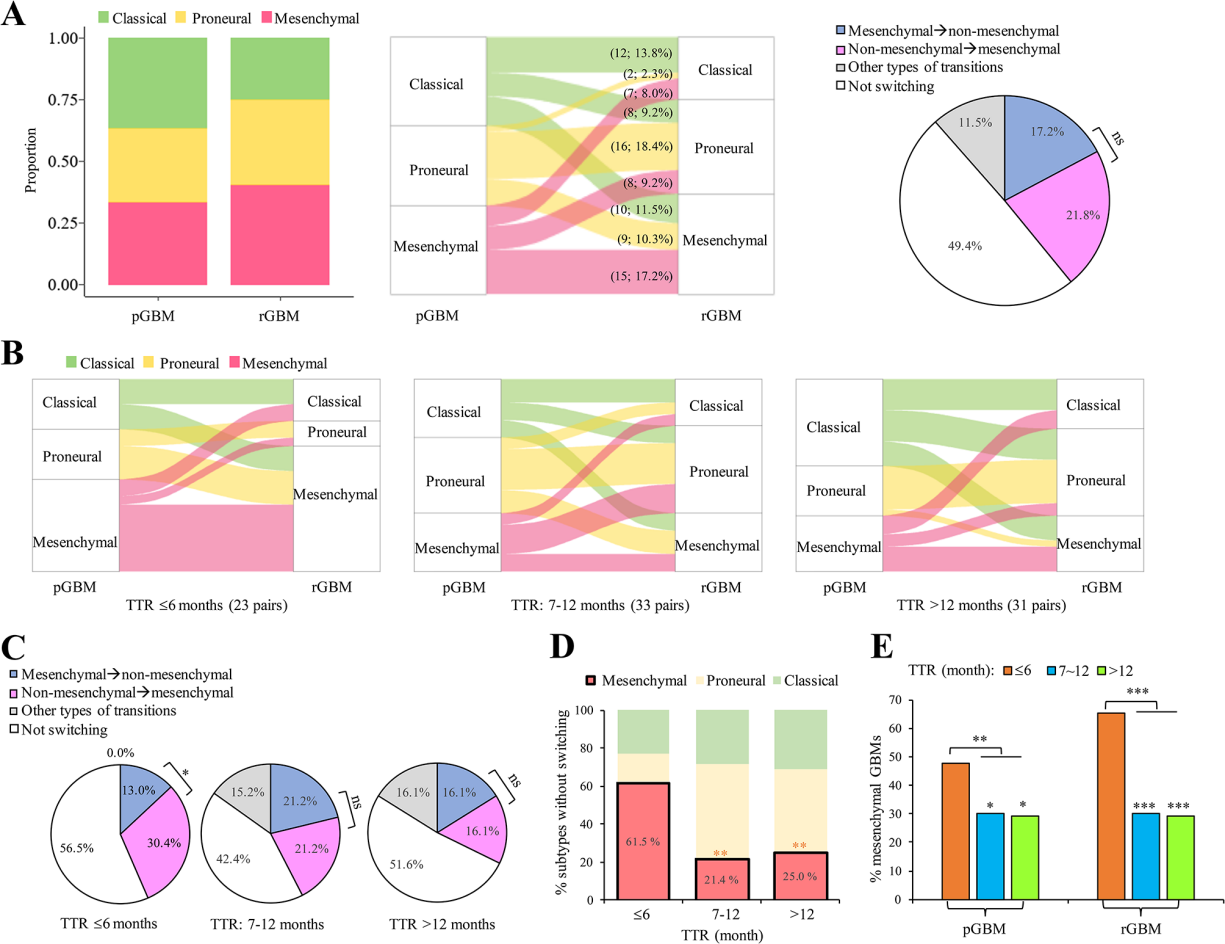
Associations of TTR with cellular heterogeneity between paired P-R GBMs. **A** Distribution of the three transcriptional subtypes (classical, proneural, and mesenchymal subtypes) in primary and recurrent GBMs. The Sankey plot and the pie chart indicated the transcriptional subtype transitions from primary GBMs to recurrent GBMs (middle) and the percentages of different subtype transitions (left), respectively. Numbers in parentheses indicated the number and percentage of types of the transcriptional subtype transitions. **B-C** Sankey plots (**B**) and pie charts (**C**) representing the transcriptional subtype changed at recurrence and the percentages of different subtype transitions with respect to TTR ≤ 6 months, TTR of 7–12 months, and TTR < 12 months, respectively. **D** Distributions of the three transcriptional subtypes with respect to TTR ≤ 6 months, TTR of 7–12 months, and TTR < 12 months, when the transcriptional subtypes remained at both time points. **E** Percentages of mesenchymal samples in primary and recurrent GBMs with respect to TTR ≤ 6 months, TTR of 7–12 months, and TTR < 12 months. For (**A**) right, (**C**), and (**E**), *P* values were evaluated using one proportion Z-Test. For (**D**) *P* values were evaluated using two-tailed Fisher’s exact test. pGBM, primary GBM. rGBM, recurrent GBM. *, *P* ≤ 0.05. **, *P* ≤ 0.01. ***, *P* ≤ 0.001. ns, not significant

We assessed the representation of three major bulk transcriptional subtypes (i.e., classical, proneural, and mesenchymal subtypes [33]) across the GLASS primary and recurrent GBMs. Like the trend observed in the recent studies [29, 30], approximately 50% of patients underwent subtype transitions at recurrence (Fig. 3A and Additional file 1: Table S1). Of the patients undergoing subtype transitions, the non-mesenchymal-to-mesenchymal transition was the most common transition, although the percentage of such a transition was not significantly different from that of the mesenchymal-to-non-mesenchymal transition (Fig. 3A). If we examined the longitudinal transitions for the patients with different TTR values, several trends were observed (Fig. 3B and E). First, the percentages of the non-mesenchymal-to-mesenchymal transition decreased with increasing TTR values (30.4% for TTR≤6 months, 21.2% for TTR at 7–12 months, and 16.1% for TTR > 12 months; Fig. 3C). Particularly, the percentage of the non-mesenchymal-to-mesenchymal transition was significantly higher than that of mesenchymal-to-non-mesenchymal transition in patients with TTR ≤ 6 months (*P* < 0.05 by one proportion z-test; Fig. 3C). Second, we observed that approximately half (42.4-56.5%) of patients remained the same transcriptional subtypes at both time points for the three TTR groups (Fig. 3C). Of note, regarding the patient-matched longitudinal GBM samples without transcriptional subtype switching, the percentage of mesenchymal GBMs was significantly higher in patients with TTR ≤ 6 months than in those with TTR > 6 months (both *P* < 0.01 by two-tailed Fisher’s exact test; Fig. 3D). In contrast with mesenchymal samples, patients with TTR ≤ 6 months had a significantly lower percentage of proneural samples as compared with those with TTR > 6 months (both *P* < 0.01 by two-tailed Fisher’s exact test; Fig. 3D). Finally, the mesenchymal GBMs exhibited the most common transcriptional subtype in both primary and recurrent GBM samples with TTR ≤ 6 months (Fig. 3E). Regarding the patients with TTR ≤ 6 months, the reason why the percentage of mesenchymal GBMs was lower in primary GBMs than in recurrent ones may be due to the high percentage of the non-mesenchymal-to-mesenchymal transition between these two time points (Fig. 3C, left).

### Correlation between paired primary and recurrent GBMs at the transcriptomic and genomic levels

**Fig. 4:**
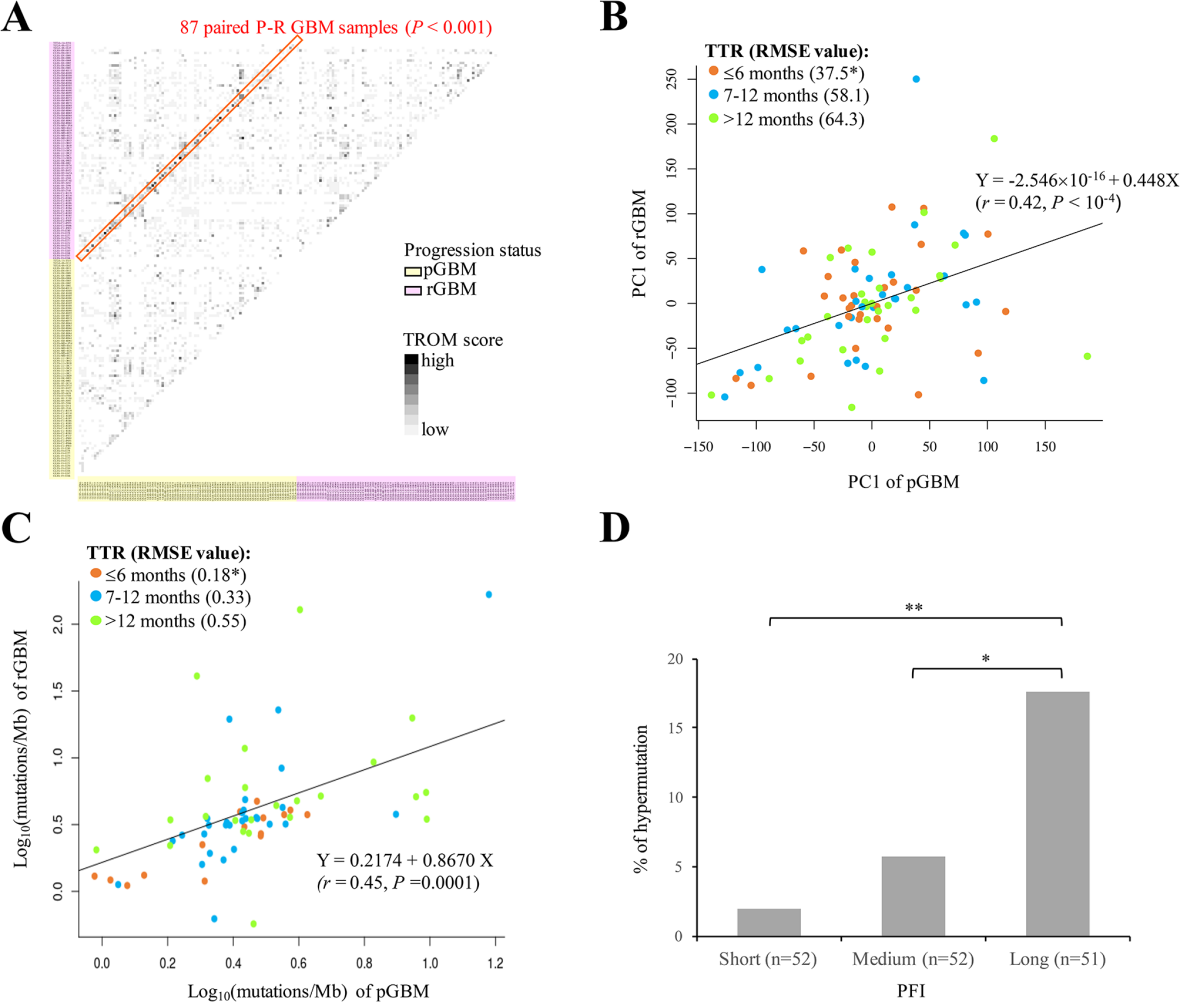
Correlation between paired primary and recurrent GBMs at the transcriptomic and genomic levels. **A** Transcriptomic similarity (measured by the TROM scores) between two GBM samples selected from the GLASS dataset (87 pGBM and 87 rGBM samples). The similarity between a sample and itself was not shown. The red box represented the 87 P-R paired GBM samples. *P* value was assessed using permutation test (see the text). **B** Correlation between the PC1s of genes expressed in paired primary and recurrent GBMs for the GLASS cohort. **C** Correlation between the TMB values (mutations/Mb) in paired primary and recurrent GBMs for the GLASS cohort. For (**B**) and (**C**), the orange, blue, and green solid dots represented the cases with TTR ≤ 6 months (23 pairs), TTR of 7–12 months (33 pairs), and TTR < 12 months (31 pairs), respectively. Numbers in parentheses indicated the fitting levels (measured by the RMSE) of the cases in the corresponding TTR groups; *P* values were assessed using permutation test (see the text). Correlation coefficients and the corresponding *P* values were evaluated using the Pearson’s correlation analysis. **D** Percentages of tumor hypermutation for the GBM patients with short (< 8.66 months; *n* = 52), medium (8.66–16.7 months; *n* = 52), and long (> 16.7 months; *n* = 51) PFIs in the G-SAM dataset. *P* values were assessed using one-tailed Fisher’s exact test. pGBM, primary GBM. rGBM, recurrent GBM. *P ≤ 0.05, **P ≤ 0.01

We then examined the correlation between gene expression profiles of paired P-R GBMs. We first asked whether the expression profiles of primary samples and their matched recurrent ones were highly similar. We evaluated the transcriptomic similarity between GBM samples using TROM scores [37] and found a significantly higher level of transcriptomic similarity for the paired P-R GBMs than for the paired samples randomly selected (*P* < 0.001 by permutation test; see Methods) (Fig. 4A). Next, for each pair of P-R GBMs, we performed PCA and determined the PC1s of all genes for the primary and recurrent GBMs, respectively (Additional file 3: Table S3). Pearson’s correlation test revealed a significantly positive correlation between the PC1s of genes expressed in these two time points (*r* = 0.42, *P* < 0.0001). Correspondingly, the linear regression equation (Y=-2.546 × 10^− 16^+0.448X) with a fitting line was conducted to fit the observed data from the paired P-R GBMs of the GLASS cohort (Fig. 4B). Intriguingly, on the basis of the fitting line, we found that the PC1s of the cases with TTR ≤ 6 months had the best fitting level (or the lowest RMSE value; see Methods), followed by those with TTR at 7–12 months, and then by those with TTR > 12 months (Fig. 4A). Particularly, the cases with TTR ≤ 6 months had a significant low RMSE value (*P* < 0.05 by permutation test; see Methods). In addition to the bulk RNA-seq data from the GLASS cohort, we integrated single-cell RNA-seq data spanning 13 paired P-R IDH-wt GBMs from an independent external cohort [38] (Methods). Although the sample size was small (*n* = 13), the similar trend of the better fitting level in cases with a short TTR than in those with a long TTR was observed (Additional file 5: Fig. S1).

Since the previous results showed that the level of genomic differences between paired P-R GBMs was positively correlated with TTR (Fig. 2C), we asked whether the TMB values of the P-R pairs were highly correlated with each other. As expected, Pearson’s correlation test revealed a significantly positive correlation between the TMB (i.e., mutations/Mb) of the cases at these two time points (*r* = 0.45, *P* < 0.001). To fit the observed data of TMB, we constructed a linear regression equation (Y = 0.2174 + 0.867X) as well as a fitting line and found that the fitting levels of the observed data generally decreased with increasing TTR values (or the RMSE values increased with increasing TTR values) (Fig. 4C). We also observed that the cases with TTR ≤ 6 months had a significant low RMSE value (*P* < 0.05 by permutation test). Regarding an independent dataset extracted from Hoogstrate et al.’s study [30] (the G-SAM cohort), we evenly divided the GBM cases into three groups according to the PFIs. Consistently, the percentages of tumor hypermutation, which was defined if at least 10 coding mutations were gained and the mutation burden of the recurrent tumor had more than 10 coding mutations per targeted Mb on the panel [30], increased with increasing PFIs (Fig. 4D). Taken together, these results reflected our above observations that both transcriptomic and genomic differences between paired P-R GBMs increased with increasing lengths of elapsed time to recurrence.

### Identification of TTR-associated molecular subtypes in primary GBM patients

**Fig. 5:**
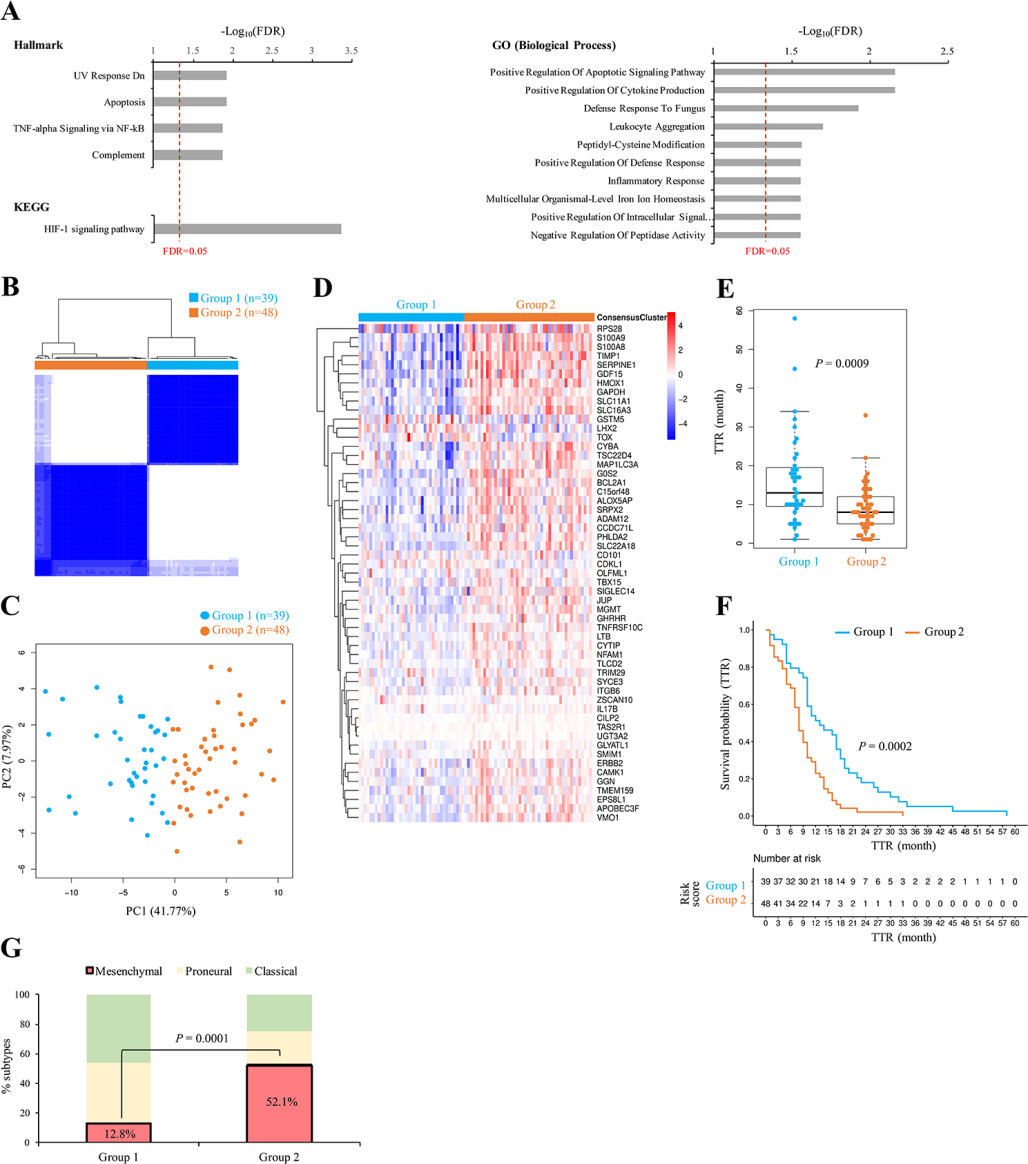
Analysis of the identified TTR-associated genes. **A** Functional enrichment analysis of the 55 TTR-associated genes using Hallmarks, KEGG pathway, and GO biological process gene sets. The *P* values were FDR-adjusted using Benjamini-Hochberg correction. The red dashed line indicates the FDR = 0.05. **B** Consensus clustering of patients with primary GBM (87 cases) using the 55 TTR-associated genes. The patients were effectively divided into two groups. **C** Principal component plots of gene expression profiles of the 55 TTR-associated genes in the 87 primary GBM samples. **D** Heatmap showing the differences in expression of the 55 TTR-associated genes in the two groups. **E** Comparison of the TTR values of the cases in the two groups. **F** Kaplan-Meier analysis of PFS for the two groups in the 87 primary GBM samples. **G** Distributions of the three transcriptional subtypes with respect to the two groups in the primary GBM samples. For (**A**) right, (**C**), and (**E**), *P* values were evaluated using one proportion Z-Test. For (**D**) and (**G**), *P* values were evaluated using two-tailed Fisher’s exact test. For (**E**) and (**F**), *P* values were evaluated using two-tailed Wilcoxon rank-sum test and log-rank test, respectively

The above results showed: (i) a lower level of transcriptomic and genomic differences in cases with a short recurrence interval than in those with a long recurrence interval; (ii) a positive correlation between paired P-R GBMs in gene expression profiles and TMB; and (iii) following the second point, such a positive correlation had a much better fit to the samples with a short recurrence interval than to those with a long recurrence interval. We thus hypothesized that the genes with similar expression patterns between the paired P-R GBM samples of a short recurrence interval (e.g., TTR ≤ 6 months) might play a role in affecting TTR. Accordingly, a protein-coding gene was identified to be a TTR-associated gene if it simultaneously satisfied the following criteria: (i) the gene was not expressed significantly differentially between paired P-R GBMs in the patients with TTR ≤ 6 months; (ii) the gene expression levels of the gene in the paired P-R GBMs were highly correlated with each other; and (iii) the gene passed the Kaplan-Meier and univariate Cox proportional hazards regression analyses with both *P* values < 0.05 (see Methods). Accordingly, 55 TTR-associated protein-coding genes were identified (Additional file 2: Table S2). Gene set enrichment analysis based on the Hallmarks gene sets from the molecular signature database [45] showed that the 55 genes were associated with cell stress/death signaling pathways (UV response and apoptosis), TNF-alpha signaling via NF-kB, and the complement system (Fig. 5A, top left). KEGG pathway analysis indicated that these genes were over-represented in HIF-1 signaling pathway (Fig. 5A, bottom left). GO analysis revealed that they were significantly enriched in the biological process terms related to apoptosis signaling pathway, inflammatory response-related biological processes (e.g., cytokine production and leukocyte aggregation), and so on (Fig. 5A, right). It is widely known that the complement system plays an important role in the maintenance of glioma stem-like cells [46], which have the potential to develop varied tumor cell populations and thereby acquire therapeutic resistance [47]. Consistently, HIF-1 signaling pathway is associated with the hypoxia-mediated maintenance of stem cells and malignant cell behavior in GBMs [48, 49]. In addition, inflammation is an important feature of tumors [50]. Inflammatory responses are often relevant to tumor invasion/metastasis and the results of radiotherapy in GBMs [51]. The response to TNF signaling is known to be important during peripheral organ inflammation in human brains [52]. TNF-alpha was reported to be essential for GBM progression [53].

On the basis of the 55 TTR-associated genes, we performed the ConsensusClusterPlus analysis [41] and precisely divided primary GBM patients (87 cases) into two different groups (Group 1 and Group 2), in which Group 1 and Group 2 accounted for 39 and 48 cases, respectively (Fig. 5B and Additional file 4: Table S4). PCA based on the 55 TTR-associated genes showed that these two groups of primary GBM patients could be grouped into separate clusters (Fig. 5C). The heatmap further revealed that these two groups exhibited a clear difference in mRNA expression, in which the 55 genes were more highly expressed in the Group 2 cases than in the Group 1 ones (Fig. 5D). Importantly, we found that the median TTR value of the Group 1 cases was significantly higher than that of the Group 2 cases (*P* < 0.001 by two-tailed Wilcoxon rank-sum test; Fig. 5E). Consistently, the Kaplan-Meier analysis suggested that patients in Group 1 had a better outcome of PFS as compared with those in Group 2 (*P* < 0.001 by log-rank test; Fig. 5F). Mesenchymal GBMs were reported to have a short TTR [29]. Indeed, the percentage of mesenchymal GBMs was significantly lower in the Group 1 cases than in the Group 2 cases (*P* < 0.001 by two-tailed Fisher’s exact test; Fig. 5G). These results revealed the significant difference in TTR between primary GBM samples in these two groups, suggesting the potential role of the TTR-associated genes for predicting TTR of primary GBMs.

### Construction of a prognostic model for TTR prediction

**Fig. 6:**
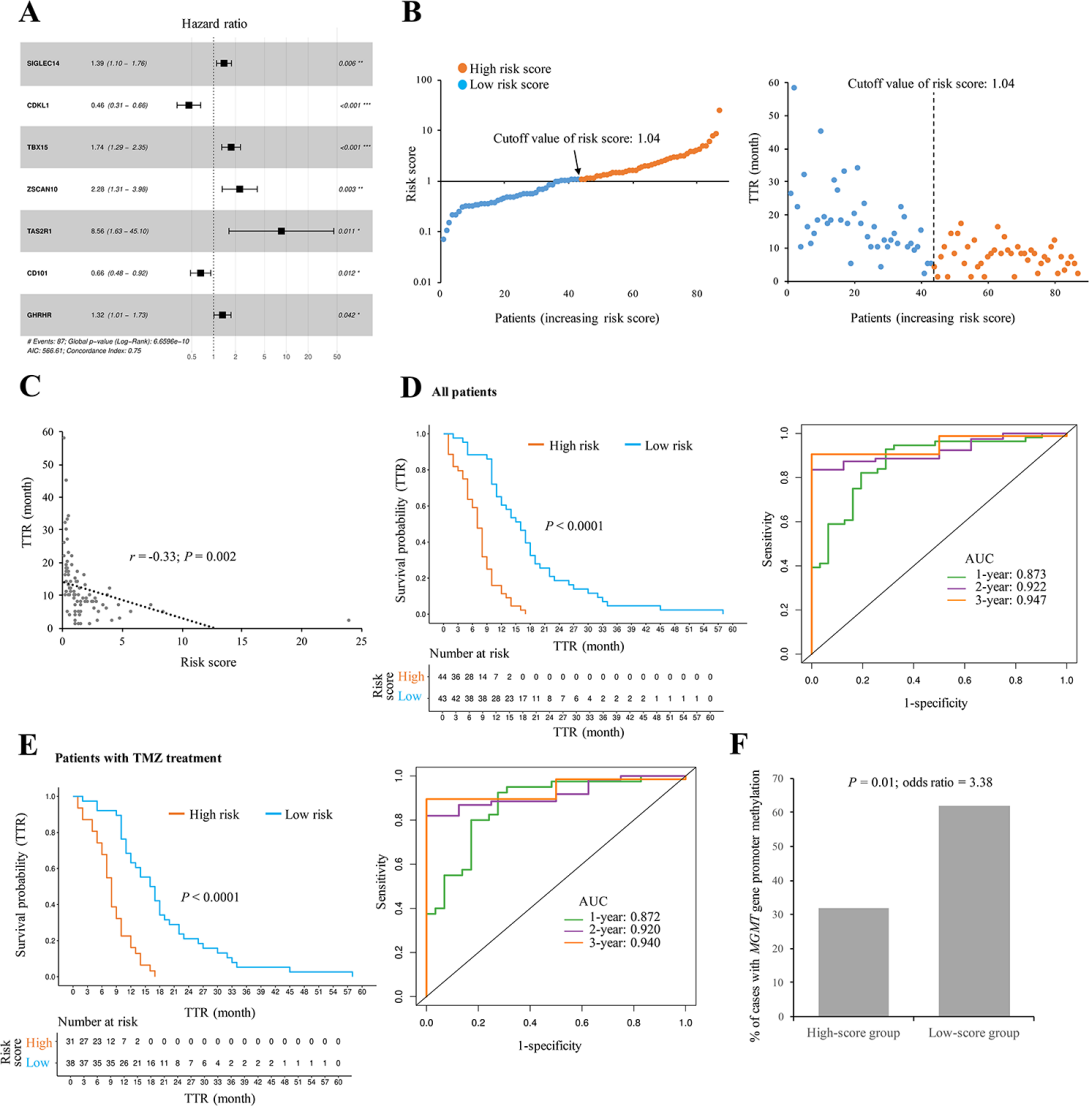
Construction of a seven-gene prognostic classifier for PFS prediction of primary GBM patients using the 55 TTR-associated genes. **A** Forest plot representing the multivariate Cox regression analysis of the seven key genes used in the constructed model. **B** Distribution of the risk scores of the prognostic model in each patient and the corresponding TTR value. The arrow indicated the median cutoff value of risk scores (1.04). **C** Correlation between the TTR values and the risk scores. **D** Evaluation of the efficiency (the Kaplan-Meier analysis; left) and accuracy (the ROC analysis; right) of the constructed prognostic model for PFS prediction of all the examined patients in the GLASS cohort (*n* = 87). **E** Evaluation of the efficiency and accuracy of the model for PFS prediction of the patients with TMZ treatment in GLASS (*n* = 69). **F** Comparison of the percentages of patients with *MGMT* gene promoter methylation for the patients with TMZ treatment in the high-score (risk score ≥ 1.04; *n* = 22) and low-score (risk score < 1.04; *n* = 21) groups. For (**A**), (**D**), and (**E**), *P* values were evaluated using log-rank test. For (**C**), correlation coefficient and *P* value were evaluated using the Pearson’s correlation analysis. For (F), *P* value were evaluated using one-tailed Fisher’s exact test

We have shown the significance of the 55 TTR-associated genes in regulating the progression of GBMs. According to these 55 genes, we performed the multivariate Cox regression analysis to construct a gene prognostic classifier for predicting TTR (or PFI). Herein, we identified seven robust prognostic markers (*SIGLEC14*, *GHRHR*, *TAS2R1*, *CDKL1*, *ZSCAN10*, *TBX15*, and *CD101*) that were independent factors significantly correlated with the TTR of the primary GBM samples (Fig. 6A). Among these seven genes, highly expressed *CDKL1* and *CD101* can server as protective factors to promote the PFS of GBM patients, whereas the high expression levels of the other five risk factors exhibited the reverse trend (Fig. 6A and Additional file 5: Fig. S2). A risk score formula based on the seven key genes was thus developed and employed to score the prognostic risk of each primary GBM patient (Methods and Additional file 4: Table S4). We dichotomized the GBM patients into high- and low-score groups using the median cutoff value of risk scores (1.04; Fig. 6B). The distribution of risk scores and TTR revealed that patients with high risk scores tended to have a shorter recurrence interval compared with those with low risk scores (Fig. 6B). Indeed, the risk scores were significantly negatively correlated with the TTR values (*P* = 0.002 by Pearson’s correlation test; Fig. 6C). Kaplan-Meier analysis revealed that the PFS rate of the high-score group was significantly lower than that of the low-score one (*P* < 0.0001 by log-rank test; Fig. 6D, left). The ROC analysis also showed that this prognostic model exhibited good predictive power with 1-, 2-, and 3-year AUC values for 0.873, 0.922, and 0.947, respectively (Fig. 6D, right). We found that approximately 80% of the GBM patients (69 out of 87 cases) in the GLASS cohort had been treated with TMZ. Regarding the patients with TMZ treatment, similar results were observed (Fig. 6E). Of note, *MGMT* gene promoter methylation is often considered as a biomarker in predicting a favorable outcome in GBM patients who are exposed to alkylating agent chemotherapy [7, 54]. Consistently, of the patients with TMZ treatment, the low-score group had a significant higher percentage of patients with *MGMT* gene promoter methylation as compared with the high-score group (*P* = 0.01 by one-tailed Fisher’s exact test; Fig. 6F).

A recent report indicated that *MGMT* gene promoter methylation status and extent of resection were important factors in affecting the patient outcome of prognostic model for PFS prediction [27]. By simultaneously considering the identified prognostic index (risk score), *MGMT* methylation status, and extent of resection, we performed a multivariate Cox regression analysis to evaluate the contribution and independence of these three features for PFS prediction in primary IDH-wt GBM patients. The multivariate Cox regression analysis showed that only the seven-gene prognostic index was significantly associated with PFS and acted as an independent prognostic factor for PFS prediction (Table 1). Taken together, these results supported the satisfactory prediction efficiency of the constructed prognostic model for predicting PFS in primary IDH-wt GBM patients.

**Table 1:** Univariate and multivariate Cox regression analyses of the risk score based on the constructed prognostic model and the two clinical features: *MGMT* methylation status and extent of resection

Variable^1^	Univariate analysis^2^				Multivariate analysis^3^		
	HR	95% CI	*P* value		HR	95% CI	*P* value
Risk score	3.352	2.063–5.449	1.05e-06		5.336	1.510-18.854	0.009
*MGMT* methylation status	3.207	1.657–6.206	0.0005		3.103	0.8963–10.744	0.074
Extent of resection	1.099	0.589–2.049	0.767		1.216	0.468–3.162	0.688

^1^For the variables of risk score, MGMT methylation status, and extent of resection, “low risk score”, “methylated”, and “total” were used as the references, respectively

^2^For risk score, 87 cases were examined (43 with a low-risk score and 44 with a high-risk score). Regarding the availability of the examined clinical features, 49 (22 with a methylated status and 27 with a unmethylated status) and 41 (22 with a total resection and 19 with a subtotal resection) cases were used for assessing the importance of the MGMT methylation status and the extent of resection in the univariate analysis, respectively. HR, Hazard ratio

^3^With having overlapping gene expression data and the two clinical features, 22 cases were examined in the multivariate analysis

### Verification of the prognostic model in independent external cohorts

**Fig. 7:**
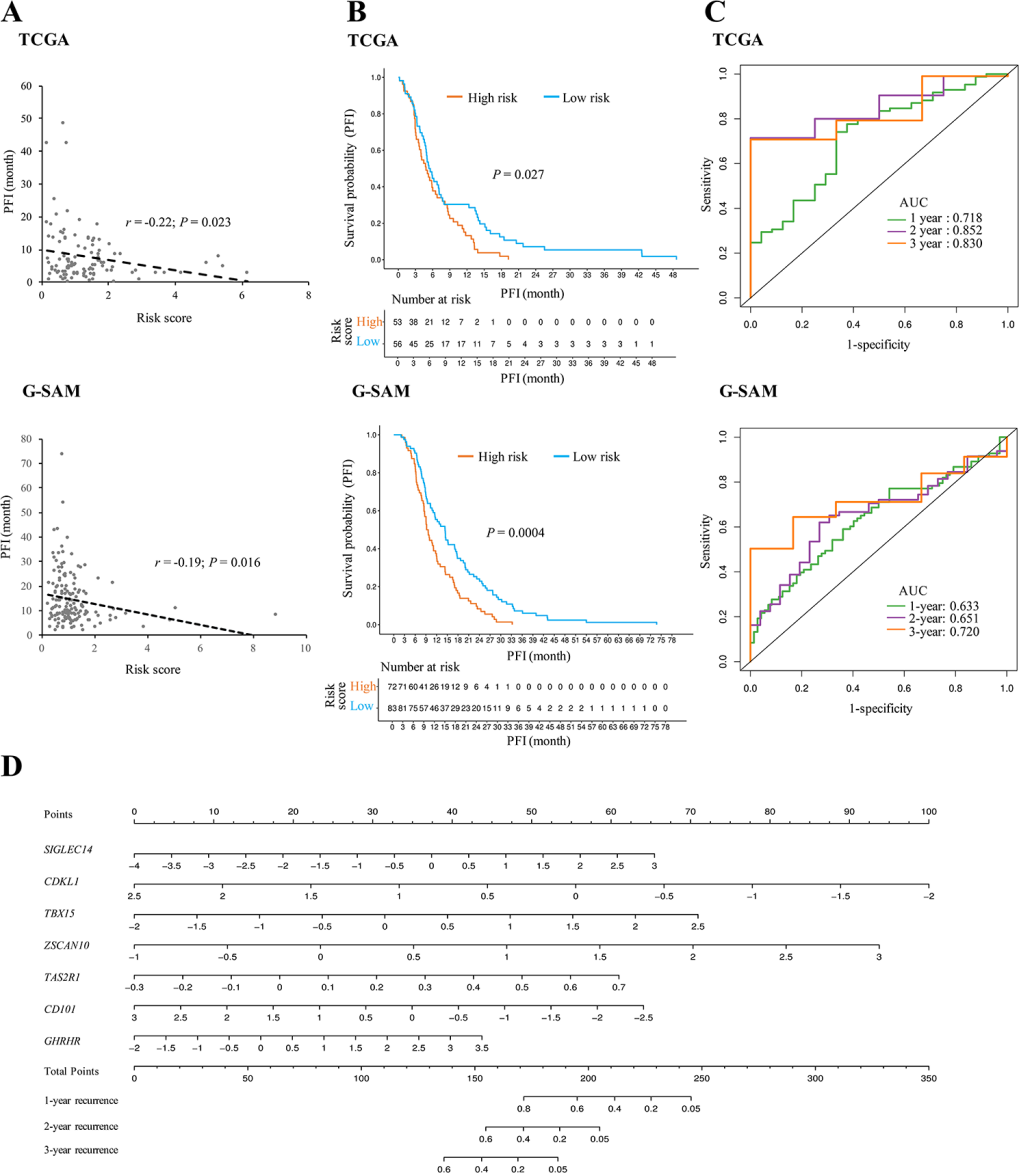
Verification of the constructed prognostic model for PFS prediction in two testing sets (the TCGA and G-SAM datasets). **A** Correlation between the PFI values and the risk scores estimated by the constructed model for the primary IDH-wt GBM cases in the TCGA and G-SAM cohorts. **B** Kaplan-Meier analyses of PFS for the groups with low- or high-risk scores in the TCGA and G-SAM cohorts. **C** Time-dependent ROC analyses of 1-, 2-, and 3-year PFS for the constructed model in the TCGA and G-SAM cohorts. **D** Construction of a nomogram for quantitatively predicting 1-, 2-, and 3-year PFS of primary IDH-wt GBM patients

Next, we examined the reliability of the seven-gene survival prediction classifier for the primary IDH-wt GBM patients from two independent external cohorts (the TCGA and G-SAM datasets), which respectively consisted of 109 and 155 IDH-wt GBM cases with complete information of sex, age, data source, and PFI (Fig. 1A). Like the trend observed in the training set (i.e., the GLASS cohort), the risk scores estimated by the constructed model were significantly negatively correlated with the PFI values in these two testing sets (i.e., TCGA and G-SAM cohorts; Fig. 7A). According to the same cutoff value of risk scores (1.04) used in the training set, we dichotomized the primary IDH-wt GBM patients of the two testing sets into high- and low-score groups, respectively. Consistently, Kaplan-Meier analyses showed a significantly lower PFS rate in the high-score group than in the low-score one (Fig. 7B). ROC analyses also indicated fair predictive power at one, two, and three years in the two testing sets (Fig. 7C). These observations supported the promising predictive performance of the constructed model for assessing the PFI of primary IDH-wt GBM patients.

Finally, we constructed a seven-gene nomogram model for clinically practical usability according to the GLASS dataset (Fig. 7D). The corresponding calibration plots were provided (Additional file 5: Fig. S3). On the basis of the nomogram model, researchers can measure the risk score of each maker gene and the total risk score for each patient according to the corresponding gene expression of the patient. Since we had demonstrated that the TTR values significantly decreased with increasing risk scores (Fig. 6C), the constructed model provided a good measurement to assess the potential time to recurrence for IDH-wt GBM patients at the primary status.

## Discussion

The GLASS dataset [29] has brought an unprecedented opportunity for investigating the evolutionary trajectories of paired P-R GBMs in terms of heterogeneities of gene expression, TMB, and microenvironment. Here we focused on studying the association of such heterogeneities with TTR. By integrating TTR with the corresponding longitudinal transcriptomic and genomic data of paired P-R IDH-wt GBMs, three major observations were unveiled in this study. First, the levels of differences between paired P-R GBMs were highly correlated with the TTR at both the transcriptomic and genomic levels. Second, both the gene expression profiles and TMB of paired P-R GBMs were highly correlated with each other. Third, on the basis of such a P-R correlation, the fitting levels of the conducted linear regression formula to the examined cases were highly associated with the TTR.

For the first observation, we found that the levels of differences for paired P-R GBM samples were significantly lower in the patients with a short recurrence interval than in those with a long recurrence interval at both the genomic and transcriptomic levels (Fig. 2). At the genomic level, we found no significant difference in TMB among the primary GBMs with different TTR, whereas the recurrent GBMs with a longer TTR exhibited a higher TMB (Fig. 2C and Additional file 5: Fig. S4). These results suggested that the positive correlation between the genomic differences between paired P-R GBM samples and TTR values was due to the longitudinal mutation accumulation during the time to recurrence. This also reflected the time-dependence of the activities of distinct mutational processes [44]. Somatic mutations were previously reported to be correlated with gene dysregulation of multiple genes in GBMs [55], suggesting that longitudinal mutation accumulation might lead to longitudinal changes of expression patterns during GBM progression. For the second observation, we found a high correlation between the paired P-R GBMs in terms of both the gene expression profiles and TMB (Fig. 4). Such a high correlation with respect to TMB (mutation counts) was also previously shown in an independent dataset from Korber et al.’s study, which consisted of 16 paired P-R IDH-wt GBM samples [19]. By extracting the corresponding gene expression data from Korber et al.’s study, we also calculated the PC1s of all genes for the primary and recurrent GBMs, respectively. Similarly, the trend of a significantly high correlation between the gene expression profiles of the paired P-R GBMs held well (Additional file 5: Fig. S5). For the third observation, on the basis of the second observation, we conducted a linear regression equation with a fitting line and found a better fitting level of the fitting line for the cases with a short recurrence interval than for those with a long recurrence interval at both the transcriptomic and genomic levels (Fig. 4). These longitudinal analyses suggested the high association of TTR with the differences between paired IDH-wt GBM tumors at both the genomic and transcriptomic levels, wherein such differences were lower in samples with a short recurrence interval than in those with a long recurrence interval.

According to the above observations, we identified 55 TTR-associated genes and demonstrated that the 55 TTR-associated genes can divide the primary GBM patients into two separate molecular subtypes, Group 1 and Group 2 (Fig. 5B and D). The subsequent analyses revealed that the Group 1 patients had a longer TTR, a better PFS, and a lower percentage of mesenchymal GBMs than the Group 2 ones (Fig. 5E and G), suggesting that the 55 TTR-associated genes might play a potential molecular typing for clinical application of IDH-wt GBMs. Through multivariate Cox regression analysis, we further constructed a seven-gene prognostic classifier for predicting TTR. Our results demonstrated the promising predictive power and robustness of the constructed model (“Model-ours”) for assessing TTR (or PFI) of primary IDH-wt GBM patients in the training and testing sets (Figs. 6 and 7). Consistently, the risk scores estimated by Model-ours were significantly lower in the Group 1 patients than in the Group 2 ones (*P* < 10^− 6^ by two-tailed Wilcoxon rank-sum test; Additional file 5: Fig. S6).

With respect to the construction of prognostic model for PFS prediction, some studies may first identify the DEGs between primary and recurrent GBM samples and then construct the prognostic models based on the DEGs [56–58]. Using this similar strategy, we also identified DEGs between the paired P-R GBMs from the GLASS dataset (Additional file 2: Table S2) and thereby constructed a new model for predicting TTR (“Model-new1”; Additional file 5: Fig. S7A). Although Model-new1 also stratified the primary GBM patients into high- and low-risk groups with a significant difference in probability of recurrence in the training set (i.e., the GLASS cohort) (*P* < 0.05 by log-rank test; Additional file 5: Fig. S7B), this prognostic model did not segregate patients from the two testing sets (the TCGA and G-SAM cohorts) into two groups with divergent PFS outcomes (both *P* > 0.05; Additional file 5: Figs. S7C and S7D). These results revealed that Model-new1 was not a robust and effective model for PFS prediction of GBM patients. On the other hand, a recent study identified genes associated with the PFI using a linear correlation method (Pearson correlation coefficient) without the information from the GBM patients at recurrence and suggested the contribution of these genes to predicting PFI [42]. We employed the same criteria to identify genes associated with the TTR and constructed another new model for predicting TTR (“Model-new2”; Additional file 5: Fig. S7E). However, similarly, the good predictive power was shown in the training set only, not in the two testing sets (Additional file 5: Figs. S7F-S7H). These results suggested that Model-new2 cannot act as a stable and effective typing tool for GBM patients. It is worthy to note that our model (Model-ours) exhibited a better model performance (a higher C-index) compared with Model-new1 and Model-new2 (Additional file 5: Fig. S7I). These results also indicated the importance of the expression patterns from the paired P-R GBMs for constructing a stable prognostic model for PFS prediction.

Regarding the constructed prognostic model (Model-ours), it consisted of seven prognostic markers, *ZSCAN10*, *SIGLEC14*, *GHRHR*, *TBX15*, *TAS2R1*, *CDKL1*, and *CD101*. Our model suggested that highly expressed *CDKL1* and *CD101* were associated with a longer TTR of GBM patients, whereas the high expression of *SIGLEC14*, *GHRHR*, *TAS2R1*, *ZSCAN10*, and *TBX15* exhibited the reverse trend (Fig. 6A and Additional file 5: Fig. S2). Of these maker genes, *ZSCAN10* is known to be important for the pluripotency of embryonic stem cells through regulating two pluripotency markers, *OCT4* and *SOX2* [59–61]. Particularly, *OCT4* was demonstrated to induce the neurosphere formation of glioma stem cells [62], contributing to GBM recurrence and resistance to radiotherapy and chemotherapy. *SIGLEC14* was found to be associated with the elevation of an inflammatory response through activating the MAPK pathway [63]. Such inflammatory mediators could play a critical role in establishing an immunosuppressed microenvironment, leading to preserving the stemness of GBM cells [64]. *GHRHR* was reported to contribute to drug resistance of sermorelin [65]. Sermorelin can inhibit the cell growth of GBM cells by penetrating the blood-brain barrier easily and was considered an effective drug for treatment of recurrent glioma patients [66, 67]. In addition, it is known that recurrent GBMs are highly immunosuppressive [68]. *TBX15* was suggested to be associated with immune cell infiltration and immunosuppression and exhibit poor clinicopathological characteristics and survival prognosis in glioma patients [69]. For the remaining signatures (*TAS2R1*, *CDKL1*, and *CD101*), the relevance of them to GBM recurrence is still greatly unknown. As potential markers and therapeutic targets for GBM patients, it is worthwhile to further investigate their biological role and pathological mechanism in glioma at recurrence.

In terms of longitudinal cellular heterogeneity, we observed that the cellular heterogeneity between primary and recurrent GBMs varied among different TTR. The patients with a short recurrence interval had a higher percentage of mesenchymal subtype compared to those with a long recurrence interval. For GBMs at recurrence, particularly, the patients with TTR ≤ 6 months were twice the percentage of mesenchymal subtype of those with TTR > 6 months (65% vs. ∼30%; Fig. 3E), in accord with a recent report that mesenchymal GBMs tended to exhibit a short surgical interval [29]. Consistently, regarding the constructed prognostic model for PFS (i.e., Model-ours), the risk scores were significantly higher in the mesenchymal GBMs than in the other subtypes (Additional file 5: Fig. S8). In addition, mesenchymal subtype was observed to be associated with a high level of myeloid cells [70], which were known to frequently emerge in the tumor microenvironment and polarize to promote tumorigenesis and immunosuppression [71]. A recent study also suggested the relevance of the distinct myeloid cell state to driving IDH-wt GBMs toward mesenchymal tumors in response to treatment [29]. By utilizing CIBERSORTx [34] based on reference cell-state signatures generated from the study of Johnson et al. [36], we deconvoluted the gene expression dataset and indeed observed a higher level of myeloid cells in recurrent GBMs than in primary ones (Additional file 5: Fig. S9). Such a trend was particularly significant for the patients with TTR ≤ 6 months (Additional file 5: Fig. S9), also reflecting our observations of a higher percentage of non-mesenchymal-to-mesenchymal transition in patients with TTR ≤ 6 months than in those with TTR > 6 months (Fig. 3C) and a higher percentage of mesenchymal GBMs with TTR ≤ 6 months in recurrent GBMs than in primary ones (48% vs. 65%; Fig. 3E).

There are several caveats in this study. Since the sample size was limited for the currently available longitudinal data from the patients with IDH-mutant GBM (see Fig. 1A), our analyses focused on the IDH-wt GBM samples. Despite the similar histology, IDH-mutant tumors are quite distinct from IDH-wt ones in prognostic and molecular features [72–74]. These two types of GBMs were also reported to undergo distinct cellular heterogeneity at recurrence [29]. It would be worthy to perform similar analyses in future work when a larger patient cohort with IDH-mutant GBM is available. In addition, different populations may exhibit considerably distinct genetic background and gene expression spectrum, leading to distinct susceptibility and progression of tumors. This underscores the requirement for applying our analysis to local and diverse cohorts with a large sample size, patient-matched longitudinal data at both the transcriptomic and genomic levels, and the corresponding clinical information (e.g., TTR or PFI) in the future. Moreover, while we have shown the promising predictive power of the constructed seven-gene prognostic model for assessing PFS of primary IDH-wt GBM patients, the mechanistic pathways of these gene markers in GBM at recurrence remain unclear and await further in-depth molecular biology investigations.

## Conclusions

In this study, on the basis of the transcriptional expression and genotype data from patient-matched longitudinal GBM samples, we observed the association of TTR with the differences between paired P-R IDH-wt GBMs at both the transcriptomic and genomic levels. According to our observations, we identified 55 TTR-associated genes and showed their potential molecular typing for clinic application of GBM patients. We thereby constructed a prognostic model with a seven-gene signature for predicting TTR (or PFI) of primary IDH-wt GBMs. The model can segregate IDH-wt GBM patients into two groups with significantly divergent progression-free survival outcomes and show effective power for predicting 1-, 2-, and 3-year PFS rates in all the training set and two independent testing sets. This study has provided helpful analysis pipeline and enlightenments for evolutionary trajectories of longitudinal GBM samples and PFS prediction of primary GBM patients.

### Electronic supplementary material

Below is the link to the electronic supplementary material.

Supplementary Material 1

Supplementary Material 2

Supplementary Material 3

Supplementary Material 4

Supplementary Material 5

## Data Availability

All data supporting the findings of this study are available within the paper and its supplementary information. The covariate-adjusted expression levels of genes for the examined IDH-wt GBM samples in the GLASS and TCGA cohorts were deposited in figshare at https://figshare.com/articles/dataset/GBM_datasets_gene_expression_by_adjusting_covariates/25051553.

## Abbreviations

AUC: Area under the receiver operating curve
DEG: Differentially expressed gene
G-SAM: The Glioblastoma, Stability of Actionable Mutations
GBM: Glioblastoma
GLASS: Glioma Longitudinal Analysis Consortium
GO: Gene Ontology
IDH: Isocitrate dehydrogenase
IDH-wt: IDH wild-type
KEGG: Kyoto Encyclopedia of Genes and Genomes
P-R: Primary and recurrent
pGBM: primary GBM
PCA: Principal component analysis
PFI: Progression-free interval
PFS: Progression-free survival
rGBM: Recurrent GBM
RMSE: Root mean square error
RNA-Seq: RNA sequencing
ROC: Receiver operating characteristic curve
TCGA: The Cancer Genome Atlas
TMB: Tumor mutation burden
TMZ: Temozolomide
TPM: Transcripts per kilobase million
TROM: Transcriptome overlap measure
TTR: Time to relapse
WHO: World Health Organization
