# Correction: A longer time to relapse is associated with a larger increase in differences between paired primary and recurrent IDH wild-type glioblastomas at both the transcriptomic and genomic levels

**DOI:** 10.1186/s40478-024-01829-5

**Published:** 2024-07-19

**Authors:** Wei-Min Ho, Chia-Ying Chen, Tai-Wei Chiang, Trees-Juen Chuang

**Affiliations:** 1 Genomics Research Center, Academia Sinica, Taipei, Taiwan; 2 Ph.D. Program in Translational Medicine, National Taiwan University and Academia Sinica, Taipei, Taiwan; 3 Department of Neurology, Chang Gung Memorial Hospital, Linkou Medical Center, Taoyuan, Taiwan; 4 College of Medicine, Chang Gung University, Taoyuan, Taiwan; 5 School of Medicine, National Tsing Hua University, Hsinchu, Taiwan

**Correction: Acta Neuropathologica Communications (2024) 12:77** 10.1186/s40478-024-01790-3

Following the publication of the original article [1], the wrong figure appeared as Fig. 7; the figure should have appeared as shown below.

**Fig. 7:**
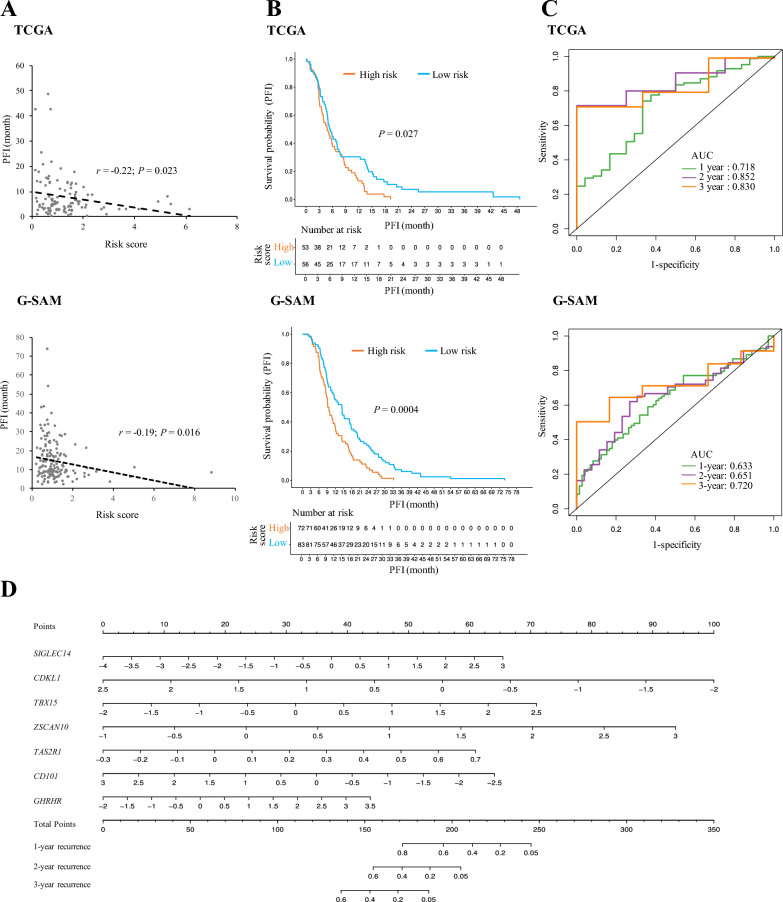
Verification of the constructed prognostic model for PFS prediction in two testing sets (the TCGA and G-SAM datasets). **A** Correlation between the PFI values and the risk scores estimated by the constructed model for the primary IDH-wt GBM cases in the TCGA and G-SAM cohorts. **B** Kaplan–Meier analyses of PFS for the groups with low- or high-risk scores in the TCGA and G-SAM cohorts. **C** Time-dependent ROC analyses of 1-, 2-, and 3-year PFS for the constructed model in the TCGA and G-SAM cohorts. **D** Construction of a nomogram for quantitatively predicting 1-, 2-, and 3-year PFS of primary IDH-wt GBM patients

The original article has been corrected.
