# Supplementary material for: A longer time to relapse is associated with a larger increase in differences between paired primary and recurrent IDH wild-type glioblastomas at both the transcriptomic and genomic levels
Source: Acta Neuropathol Commun. 2024 May 18;12:77. doi: 10.1186/s40478-024-01790-3 (PMC11102269; doi:10.1186/s40478-024-01790-3)
Supplement: Supplementary file 5 — Supplementary Material 5 [file 40478_2024_1790_MOESM5_ESM.docx]

Supplementary information for

**A longer time to relapse is associated with a larger increase in differences between paired primary and recurrent IDH wild-type glioblastomas at both the transcriptomic and genomic levels**

**Wei-Min Ho, Chia-Ying Chen, Tai-Wei Chiang****, and Trees-Juen Chuang^*^**

* Correspondence: trees@gate.sinica.edu.tw

**Figure S1.** Correlation between paired primary and recurrent GBMs in gene expression profiles based on the single-cell RNA-seq data. The orange and blue solid dots represented the cases with a long TTR and a short TTR (median of TTR = 301 days), respectively. Numbers in parentheses indicated the fitting levels (measured by the RSEM) of the cases in the three TTR groups. Correlation coefficient and *P* value were evaluated using the Pearson’s correlation analysis. pGBM, primary GBM. rGBM, recurrent GBM.

**Figure S2.** Kaplan-Meier analysis of the seven key genes (*SIGLEC14*, *GHRHR*, *TAS2R1*, *CDKL1*, *ZSCAN10*, *TBX15*, and *CD101*) in the GLASS dataset. *P* values were evaluated using log-rank test.

**Figure S3.** Calibration curves for predicting TTR at one and two years for the primary IDH-wt GBM patients. The curve for predicting TTR at three years was not plotted because of the small sample size for the patients with TTR > 3 years.

**Figure S4.** Comparisons of the TMB values (mutations/Mb) among the patients in the three TTR groups (TTR ≤ 6 months, TTR of 7-12 months, and TTR < 12 months) for primary and recurrent GBM samples. *P* values were evaluated using two-tailed Wilcoxon rank-sum test. pGBM, primary GBM. rGBM, recurrent GBM. *, *P* ≤ 0.05. **, *P* ≤ 0.01. ns, not significant.

**Figure S5.** Correlation between the PC1s of genes expressed in paired primary and recurrent GBMs (n=16) for the GBM dataset generated by Korber et al. (Korber V*, et al.* Evolutionary Trajectories of IDH(WT) Glioblastomas Reveal a Common Path of Early Tumorigenesis Instigated Years ahead of Initial Diagnosis. *Cancer Cell* **35**, 692-704 e612 (2019)) Correlation coefficients and *P* values were evaluated using the Pearson’s correlation analysis. pGBM, primary GBM. rGBM, recurrent GBM.

**Figure S6.** Comparison of the risk scores (based on the constructed model, Model-ours) of the patients in the two molecular groups illustrated in Figure 5 (Group 1 with 39 cases and Group 2 with 48 cases). *P* value was evaluated using two-tailed Wilcoxon rank-sum test.

**Figure S7.** Assessment of the predictive power of the two prognostic models, Model-new1 and Model-new2, in the training set (the GLASS cohort) and two testing sets (the TCGA and G-SAM cohorts). **A** The key genes identified by the multivariate Cox regression analyses for Model-new1. **B-D** Kaplan-Meier analyses of the progression-free survival for the two groups segregated by Model-new1 in the primary GBM samples of the GLASS (n = 87), TCGA (n = 109), and G-SAM (n=155) cohorts, respectively. **E** The key genes identified by the multivariate Cox regression analyses for Model-new2. **F-H** Kaplan-Meier analyses of the progression-free survival for the two groups segregated by Model-new2 in the primary GBM samples of the GLASS (n = 87), TCGA (n = 109), and G-SAM (n=155) cohorts, respectively. **G** Comparison of the concordance indices (C-indices) of Model-ours, Model-new1, and Model-new2. All *P* values were evaluated using log-rank test. Coef., coefficient. HR, hazard ratio.

**Figure S8.** The risk scores (based on the constructed prognostic model for PFS) of the mesenchymal, proneural, and classical subtypes of the primary GBMs in the GLASS dataset. *P* values were evaluated using two-tailed Wilcoxon rank-sum test.

**Figure S9.** Cell-state compositions of primary and recurrent IDH-wt GBM samples with TTR ≤ 6 months, TTR of 7-12 months, and TTR < 12 months. *P* values were evaluated using two-tailed Wilcoxon rank-sum test.
